# The Aboriginal Australian Family Wellbeing Program: A Historical Analysis of the Conditions That Enabled Its Spread

**DOI:** 10.3389/fpubh.2018.00026

**Published:** 2018-03-01

**Authors:** Janya McCalman, Roxanne Bainbridge, Catherine Brown, Komla Tsey, Adele Clarke

**Affiliations:** ^1^Centre for Indigenous Health Equity Research, School of Health, Medical and Applied Sciences, Central Queensland University, Cairns, QLD, Australia; ^2^The Cairns Institute, James Cook University, Cairns, QLD, Australia; ^3^Doctoral Program in Sociology, University of California, San Francisco, San Francisco, CA, United States

**Keywords:** scaling up, spread, implementation, Indigenous, health and wellbeing

## Abstract

**Introduction:**

Spreading proven or promising Aboriginal health programs and implementing them in new settings can make cost-effective contributions to a range of Aboriginal Australian development, health and wellbeing, and educational outcomes. Studies have theorized the implementation of Aboriginal health programs but have not focused explicitly on the conditions that influenced their spread. This study examined the broader political, institutional, social and economic conditions that influenced negotiations to transfer, implement, adapt, and sustain one Aboriginal empowerment program—the Family Wellbeing (FWB) program—to at least 60 geographical sites across Australia over 24 years.

**Materials and methods:**

A historical account of the spread of the FWB Program was constructed using situational analysis, a theory-methods package derived from a poststructural interpretation of grounded theory methods. Data were collected from published empirical articles, evaluation reports and project articles, and interviews with 18 key actors in the spread of FWB. Social worlds and arenas maps were used to determine the organizations and their representative agents who were involved in FWB spread and to analyze the enabling and constraining conditions.

**Results:**

The program was transferred through three interwoven social arenas: employment and community development; training and capacity development; and social and emotional wellbeing promotion and empowerment research. Program spread was fostered by three primary conditions: government policies and the availability and Aboriginal control of funding and support; Aboriginal leadership, associated informal networks and capability; and research evidence that built credibility for the program.

**Discussion and conclusion:**

The continued demand-driven transfer of empowerment programs requires policies that enable Aboriginal control of funding and Aboriginal leadership and networks. Flexible and sustained coordination of program delivery is best leveraged through regional innovation hubs that can work with partner organizations to tailor the program to local end-user needs. Associated research is also needed to evaluate, continually improve program quality, and build program credibility through evidence.

## Introduction

Decision-makers in the developed settler-colonized countries of Canada, Australia, New Zealand and the United States (CANZUS nations) struggle to discern how to best close the gaps in Indigenous and non-Indigenous health equity. Enabling the spread of proven programs can make potentially cost-effective contributions to a range of Indigenous development, health and wellbeing, and educational outcomes (1). There is great interest in this field in CANZUS nations; yet reviews of Indigenous health implementation evidence (1–3) have found limited literature. Individual studies have explored Indigenous people’s understandings of what is important in implementation. They suggest that effective implementation is a product of the efforts of individual change agents to spread proven approaches, and require favorable political, institutional and economic conditions that support empowering implementation processes (4). However, studies have not explicitly explored how these structural conditions enable or impede program spread over time, or how conditions could be ameliorated to better facilitate the spread of Indigenous health programs.

A number of different terms are used in the international literature to describe the processes of spreading programs and services. Terms such as transfer, translation, exchange, diffusion, dissemination, implementation, scaling, utilization, uptake, and linkage are often used interchangeably. The term “scaling” is used in this article to refer to the composite processes by which a program or service: (1) is spread to a new setting (transferred), (2) is assimilated and changed in the new organizational setting (implemented), (3) develops endurance over time (sustainability), (4) creates transfer of knowledge and authority from the external provider to the new organization (shift in ownership and capacity), and (5) is appropriately revised by those adapting it (adaptation) (5). The term program is used to refer to a packaged system of services that work together to produce impacts for individuals or communities (6).

International evidence suggests that whilst many health programs are implemented effectively in single communities, there are considerable barriers to scaling them up across a nation. Barriers include high economic costs of the intervention or implementation per participant; adverse political or institutional barriers; differing values of funding bodies and local end-user organizations, lack of clear roles or functions, or lack of compatible incentives; lack of adaptation to the local context; and lack of scaling-up logistics (7). The key enabling conditions for scaling up are strong political commitment from governments to create and nourish a lively and empowered civil society and strong community representation; and well-designed political, administrative, and economic decentralization (7). The availability of resources, particularly funding from government programs is critical to program scaling, particularly the sustainability of implementation (8).

In this study, we examined the scaling of the Aboriginal-developed Family Wellbeing (FWB) Program, that spread nationally to 60 known sites and was delivered to more than 3,500 participants through a minimum of 220 episodes over 24 years (9). A prior grounded theory study described how Aboriginal Australians and allied others were strongly motivated to scale up the program across these multiple sites as a vehicle for Aboriginal-developed working toward their own empowerment and to support the empowerment of others (9, 10). The process of transfer exemplifies what is described in the international literature as an “empowering facilitated evolutionary process.” This entails informal and largely uncontrolled transfer, negotiated laterally through peer networks and brokered between organizations and funders on a situation by situation basis (11). There was significant variation across time and place associated with the number and capacity of providers, partnerships with other organizations, motivation, and structural factors such as resourcing. However, the earlier grounded theory study did not explicitly consider the broader conditions that enabled or hindered program scaling.

This study seeks to contribute to understandings of program scaling by examining the broader conditions that influenced the transfer, implementation, adaptation and sustainability of the FWB Program. These conditions included the national Australian policies for Indigenous affairs, which changed three times within the 24-year timeframe of FWB Program spread. The policies of Indigenous self-management (1975–96), mainstreaming (1996–2004), shared responsibility (2004–2014), and advancement (2014-current) provided shifting macro-level political, ideological, socioeconomic and other systems of relating, and influenced meso-level social network ties among individuals and organizations. Following Adele Clarke (12, 13), such structural conditions of Indigenous Australian health are considered to be integral within and constitutive of situations, rather than surrounding or distinct from them:

*“*There is no such thing as ‘context’”. Instead, the *“conditions of the situation are in the situation*… The conditional elements of the situation need to be specified in the analysis of the situation itself as *they are constitutive of it*, not merely surrounding it or framing it or contributing to it. They are it…” (Clarke, 2005, pp. 71–72, emphasis in original).

Hence, the research question is: *How do the broad (varied and changing) conditions of Australian as a situation make themselves consequential in efforts to scale up an Aboriginal health program?*

## Materials and Methods

### The Program

**Figure 1:**
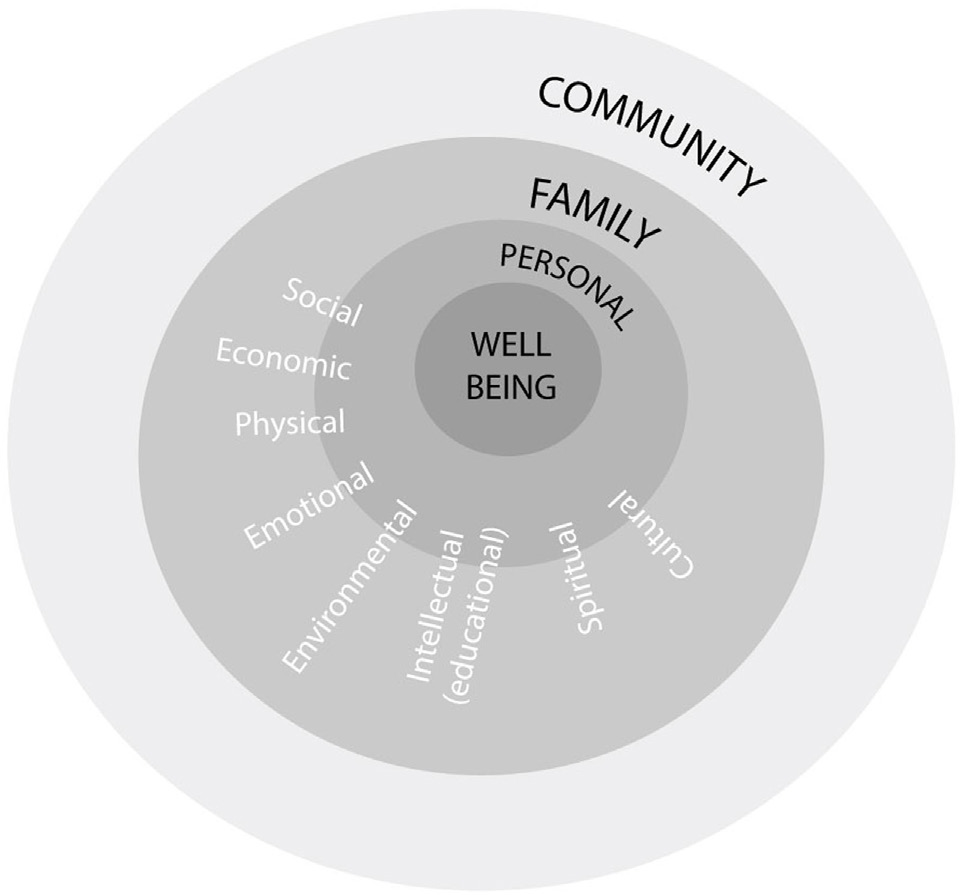
Model for promoting Aboriginal development and wellbeing. Source: adapted from Aboriginal Emptoent Development Bianch, 1994a, 1994b.

The FWB program was developed in 1993 by the Aboriginal Employment Development Branch of the South Australian Department of Education, Training and Employment (The Branch). At first glance, FWB is an accredited Certificate II training program offered through the Australian vocational education and training sector. Skills taught included foundational counseling skills for coping with personal and community problems including grief and loss. However, the complex nature of Aboriginal Australian wellbeing issues and their determinants calls for more than a standard didactic training program. FWB was therefore designed to provide an empowering framework within which participants are supported to interact and tackle a variety of personal, professional and community wellbeing issues. The program is based on theoretical models of change from psychosynthesis (14), and community development including a continual action learning cycle to reflect and act upon practices, activities and procedures; directions and purpose; and unity and identity (15, 16). The result is a comprehensive ecological program for promoting Aboriginal development and wellbeing at multiple levels (Figure 1).

Family Wellbeing facilitators support program participants to interact and reflect on the development of self, relationships with others (including family members) and relationships between groups (families, clans and the wider society). Participants are invited to consider their personal issues, blocks, and barriers to change (17). The impacts of colonization on people’s lives is acknowledged and participants are asked: “How can we heal our wounds? Who are we? Why are we here and what are our beliefs? What do we want to do and how do we want to do it?” (18). Such questions elicit participants’ reflections on their physical, emotional, mental and spiritual needs, experiences throughout their life journey, and qualities and strengths.

Relationship issues are then considered. These include communication and understanding; conflict resolution; forgiveness, reconciliation and healing; parenting skills; love and nurturing; interdependence; and other specific issues for men, women, parents or children. Finally, a participant is asked to focus on issues concerning relationships between groups including respecting and understanding differences; conflict resolution; forgiveness, reconciliation and healing; sharing, cooperation and learning from each other; and interdependence (17). Participants are supported in identifying goals for personal change, in reclaiming traditional wisdoms, and in becoming agents for change in their families and communities.

### The Research Approach

A historical account of the transfer and implementation of FWB Programs was constructed using situational analysis, a theory-methods package derived from a poststructural interpretation of grounded theory methods (12, 13). Situational analysis is used to analyze situations of inquiry through mapping tools, one of which is social worlds and arenas analysis. Social worlds are “groups with shared commitments to certain activities,” in this case, the scaling of FWB. They shared resources of many kinds to achieve their goals, and built shared ideologies about how to go about their business, generating shared identities, perspectives and ideologies that then formed the basis for negotiation of conflict, exchange of ideas, and cooperative action (19–22). Social worlds have “changing porous boundaries” and people characteristically are participants in a multiplicity of worlds (7). Social arenas result from interaction within and between social worlds that all focus on a given issue or topic area, such as Aboriginal health (21). Social arenas are constantly in flux as a result of the actions of layered mosaics of social worlds, which can act as constraints on the work of another world or provide resources or opportunities.

Maps of the social worlds and arenas were developed to determine the broad structural conditions that influenced interactions to transfer and implement the FWB Program, the arenas of commitment and discourse within which they were engaged, and the organizations or groups and their representative individual agents of change (12, 13). The sensitizing concept of the interface between Aboriginal and Western knowledge systems was used in developing the social worlds maps (23). While there are considerable differences between Aboriginal communities and no clear Aboriginal/Western knowledge dichotomy, the concept of the interface was useful for considering how Aboriginal and Western discourses and constructions of meaning intersected to shape and influence program scaling.

This study was carried out in accordance with the recommendations of James Cook University Ethics Committee with written informed consent from all subjects in accordance with the Declaration of Helsinki. The protocol was approved by the James Cook University Ethics Committee (H 3532).

### Data Collection

A phased approach was used to reconstruct the 24-year history of the transfer and implementation of FWB. First, 60 published articles and evaluation reports that provided empirical documentation of FWB implementation, were located through literature searches, networking, and enquiry with providers of FWB delivery across Australia. Project planning documents were also collected. However, there were gaps in the documented evidence. The first published program evaluation, for example, was not completed until 7 years after the genesis of FWB, and after significant program spread (24). As the study progressed, it became apparent that significant across-state and across-target group transfers had similarly gone undocumented.

Second, to fill identified gaps, information was obtained through interviews with 18 FWB agents. They included 8 Aboriginal and 10 non-Aboriginal people who were committed and engaged as program developers, coordinators, facilitators, adapters, researchers, and/or advocates for FWB scaling. As employees of organizations, all FWB agents acted for the organizations’ interests, but they also maintained some discretion to interact, advocate for program delivery and negotiate program transfer and implementation. Their involvement in transferring FWB spanned from the origination of the program in 1993 to the time of interview, encompassing their involvement in 177 of the 206 (86%) situations of FWB transfer.

Family Wellbeing agents were differentiated from FWB Program participants or students of the program, although all but three had also participated. Research respondents were chosen through purposive and later theoretical sampling to interrogate the diversity of emerging concepts across time, places and types of transfer. As recommended by ethics guidelines for Indigenous research, attention was paid throughout the research to principles of spirit and integrity, reciprocity in the research relationship, respectful relationships, equality in power relationships, survival and protection including support for a collective Indigenous identity, and responsibility to do no harm (NHMRC, 2003). The interviewer acknowledged her own positioning as being outside the Aboriginal colonized experience while at the same time seeking to be an “allied other” (25).

### Data Analysis

Social worlds and arenas maps were developed to determine the enabling and constraining conditions, organizations or groups involved in FWB transfer and their representative individual agents of change, and the extent of program scaling. Reflective memos were used to clarify the elements represented by the maps, their relations and the significance for the study. For example, an early reflective memo identified that FWB as a program intervened at the boundaries between social worlds, but was also a social world in itself. The structural conditions were identified by comparing cross-cutting themes across social arenas.

## Results

**Figure 2:**
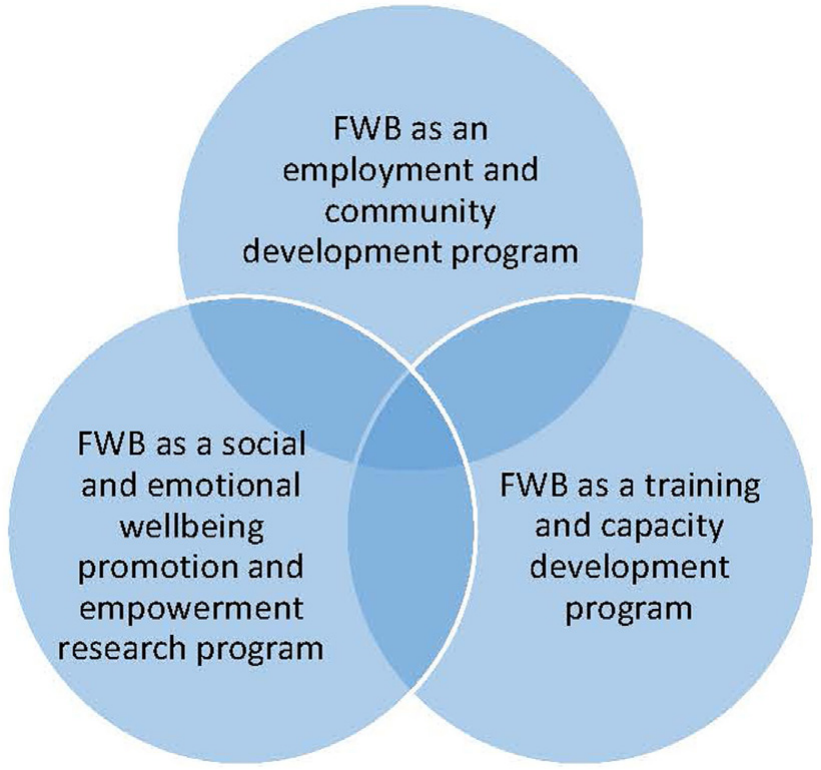
The intersecting social arenas of Family Wellbeing.

Three overlapping social arenas were identified. These were Aboriginal employment and community development (1992–1998), Aboriginal training and capacity development (1995–current), and Aboriginal social and emotional wellbeing promotion and empowerment research (1996–current) (Figure 2).

The arenas are described in the following sections, including the process by which the program was introduced into the arena, the primary intent of program delivery within the arena, key provider and partner organizations, and chronology of key events and the broader conditions, illustrated with some examples.

### Arena 1: Aboriginal Employment and Community Development (1992–1998)

**Figure 3:**
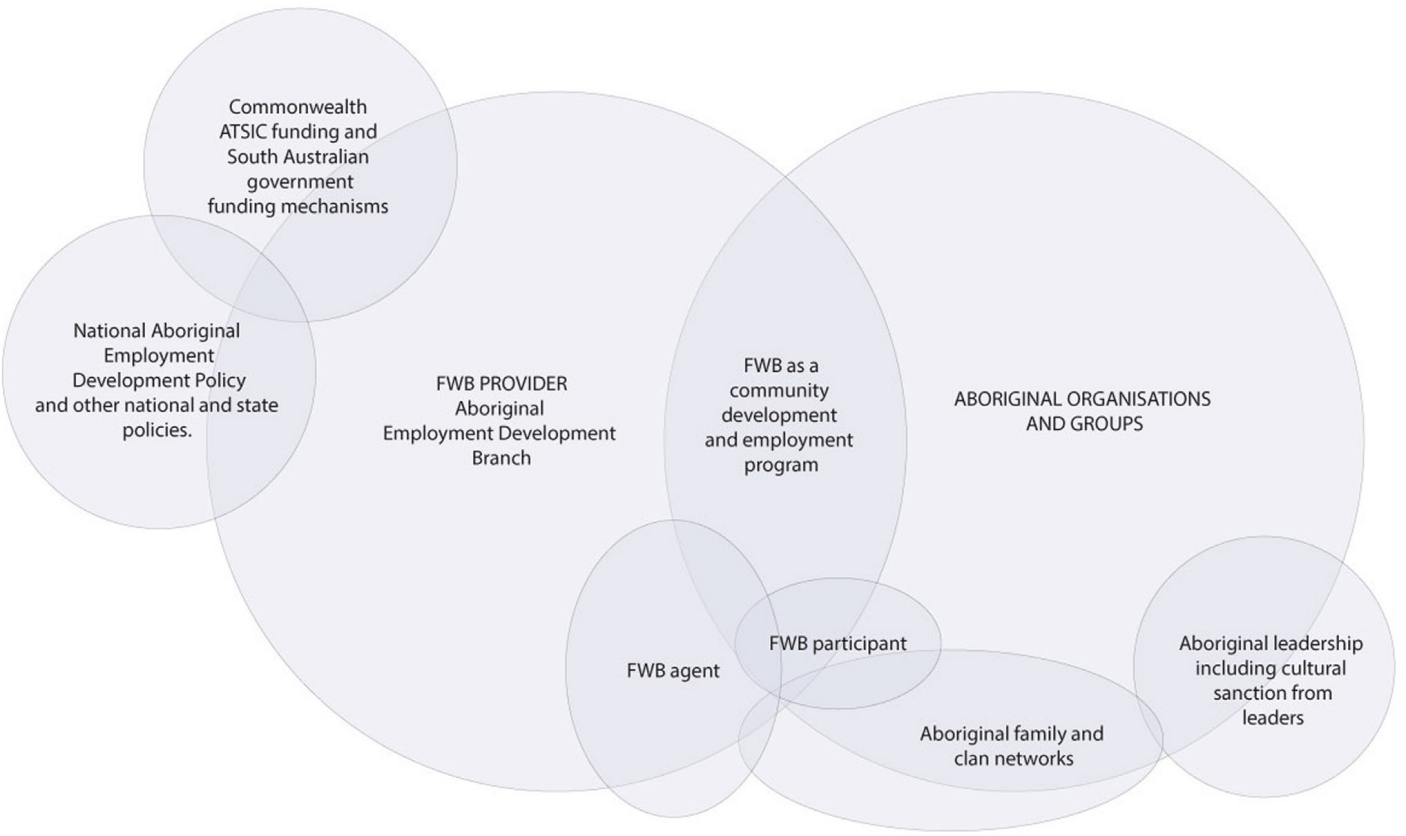
Project map of the social worlds of FWB framed through the arena of community development and employment (1992–1998).

Family Wellbeing was originally designed as an employment and community development program. Figure 3 depicts the two primary social worlds engaged in the genesis of FWB: the Aboriginal Employment Development Branch (hereafter called the Branch) of the South Australian Department of Education, Training and Employment and Aboriginal community organizations and groups. Under the umbrella of the national Aboriginal policy of self-management, which by the early 1990s had been in place in Australia for almost 20 years, the Branch was tasked with a range of strategies to comply with the provisions of the national Aboriginal Employment Development policy (26) (left of Figure 3). The policy had been introduced in 1986–1987 to increase the range of work and training opportunities for Aboriginal people at all levels of the public sector as well as to provide support for long-term Aboriginal economic development processes (27). Initial program funding was made available from the Commonwealth Aboriginal and Torres Strait Islander Commission (ATSIC) and the state government (17) (left side of Figure 3).

Family Wellbeing sits at the intersection of the Branch and Aboriginal organizations and groups (right of Figure 3). Learning from previous South Australian Aboriginal community development planning approaches that had produced a low level of community involvement, interest and commitment (28), early FWB developers recognized the importance of starting with the concerns and initiatives of Aboriginal people and facilitating change processes driven primarily through engagement with community members (29). Important community development principles included the building of Aboriginal social capital and community empowerment, leadership and participation; tackling Aboriginal people’s priority issues and finding solutions by leveraging further investments in the community; and building elements of sustainability (30, 31). The Branch therefore designed a bottom-up community development and employment approach in consultation with Aboriginal organizations and groups (right of Figure 3). This was accomplished partly through the informal and family ties (right of Figure 3) of the Branch Director, Les Nayda.

Once the program theory had been drafted, the FWB originators visited Aboriginal organizations and groups in Adelaide and regional South Australian communities to explain the program, hear their feedback and seek their engagement. First, Nayda drew again upon his Aboriginal family and clan networks, taking two of the early FWB facilitators to meet traditional Aboriginal Elders of the Pitjantjatjara lands in Central Australia, to seek cultural sanction. Upon receiving the Elders’ blessing, he set out to engage Aboriginal organizations.

Engaging organizations to implement FWB required overcoming serious historical shortcomings related to prior partnerships between Aboriginal organizations and governments. These had resulted from previous government approaches which created “distrust, enmity and disputation” because programs had not been culturally appropriate and lacked locally relevant aims, resulting in a failure to attract Aboriginal participation (27). FWB lunches were offered to community members as theirs to own and run as they wished. An early FWB document stated that “the overriding requirements are that the sessions be open, voluntary and that anyone can come on an equal basis and that the program be owned and arranged by the community with no Departmental or official interference” (17).

At one early lunch at Murray Bridge, for example, 15 Aboriginal community members of all ages met in the back garden of a local childcare center. In an open informal discussion, members revealed their sense of connection and love with family, their aspirations for health, education, contentment and satisfying relationships. They also spoke about their daily concerns and worries about their children, and problems of family violence, alcoholism, conflict, isolation and youth at risk. They decided to meet fortnightly at the center to discuss identified issues with an invited facilitator. Branch representatives were invited, but asked to leave their positions at the gate.

At the next session, twenty people came; discussing how members could nurture themselves to be better able to cope with daily frustrations and then care better for others. Significant family problems, depression, lack of purpose and alienation became evident and, at the end of the session, three of the families requested individual counseling with the facilitator who was a trained family therapist (17). This example demonstrated the central role of community members in determining their own priorities, which varied from community to community and family to family. Through FWB, the voices of Aboriginal people were privileged in order to bridge the somewhat tenuous interface between government and Aboriginal community members. An Aboriginal FWB facilitator, later reflected:

It gives that two-way understanding, that’s what FWB does…We’re all at this level of understanding… it gets back to that safe space. It allows that two-way understanding to take place because it’s creating that safe place for the dialogue to occur.

A shift in the orientation of program occurred when a participant at a community lunch in Ceduna asked about the availability of training for grief and crisis resolution: “Is there any training in this?” (research respondent, personal communication, August 6, 2010). The first stage of what was to become the FWB training program was developed in response to this request. The relevance of fore-fronting Aboriginal concerns was expressed by one early FWB facilitator:

You’re a product of past history of what happens. I guess when I stand up as an Aboriginal person and go through my life’s experiences and my childhood and teenage years, and more times out of none, every Aboriginal student in that class is going to comprehend what I’m saying because they’ve had the same journey.

The FWB training program was first delivered in 1993 at Port Augusta over a 9-week period, with a 3-h module delivered each week (27 h). Bolstered by the enthusiastic response to the training program by community groups, in 1994 the Branch developed an ambitious strategy for scaling up the program.

Unlike other community development initiatives, commonly implemented in single community settings, ATSIC funding enabled the Branch to develop short-term objectives (to June 1995) which included development of FWB centers in every major South Australian Aboriginal community. Aboriginal coordinators were to be employed and skilled to deliver an accredited FWB counseling training and other courses to train and empower members of all major Aboriginal communities. As well, resources, publicity materials and videos would be developed. The result would be a highly trained FWB team able to anticipate and respond to changing community needs and work according to a code of ethics. Longer term objectives (to June 1998 and beyond) included further program scaling and extension of each of the strategies (18).

By 1995, six South Australian centers had been established (in Port Augusta, Coober Pedy, Murray Bridge, Ceduna, Point Pearce, and Adelaide), with Aboriginal FWB workers employed in each. Their role was to organize FWB lunches designed to “bring together Aboriginal groups, families and communities to develop a common vision which promoted unity, self-responsibility and economic independence for the comprehensive development and wellbeing of each Aboriginal person, family and community” (18). Port Adelaide and Ceduna (1994), Murray Bridge and Whyalla (1995), Alice Springs (1996), and later other South Australian communities requested the training. In response to further community requests, FWB was again adapted to add stages 2–4 (a further 90 h) (32).

Additionally, the Branch organized events for training, team building and networking; three are notable. In 1994, a FWB conference was held on traditional Pitjantjatjara land in northern South Australia to provide an opportunity to enhance the connectedness of the growing FWB networks with Aboriginal family groups. It attracted approximately 100 participants and media attention. Soon afterward, a challenging personal and professional development workshop was provided for the incipient FWB facilitators and others by a visiting American psychosynthesis therapist, Edith Stauffer (33). In early 1996, the Branch also coordinated a well-received 12-week cross-cultural exchange visit by Tibetan Gyoto Buddhist monks to five remote Aboriginal communities (32).

By 1996, however, broader national debates about Australia’s welfare system, including options for reducing Aboriginal welfare dependency, started to impact the FWB approach. Despite public Commonwealth and state government statements of commitment to Aboriginal empowerment, partnership and reconciliation, from 1996 on, after the national election of the conservative Howard Government: “it was clear that he intended to undo much that he had inherited in the Indigenous Affairs portfolio” (34). A new national policy of mainstreaming Aboriginal-specific programs led to budget cuts for the FWB Program and the Branch’s capacity to responsively implement the program according to community demand waned dramatically.

Still far short of achieving their goal of FWB centers in every major South Australian Aboriginal community, by 1999 most of the original FWB agents had resigned, and had been replaced by a succession of short-term leaders. The employment of the regional FWB workers could not be sustained, and the community lunches were phased out. After 1998, FWB was no longer explicitly framed as a community development and employment program.

### Arena 2: Aboriginal Training and Capacity Development (1995–Current)

**Figure 4:**
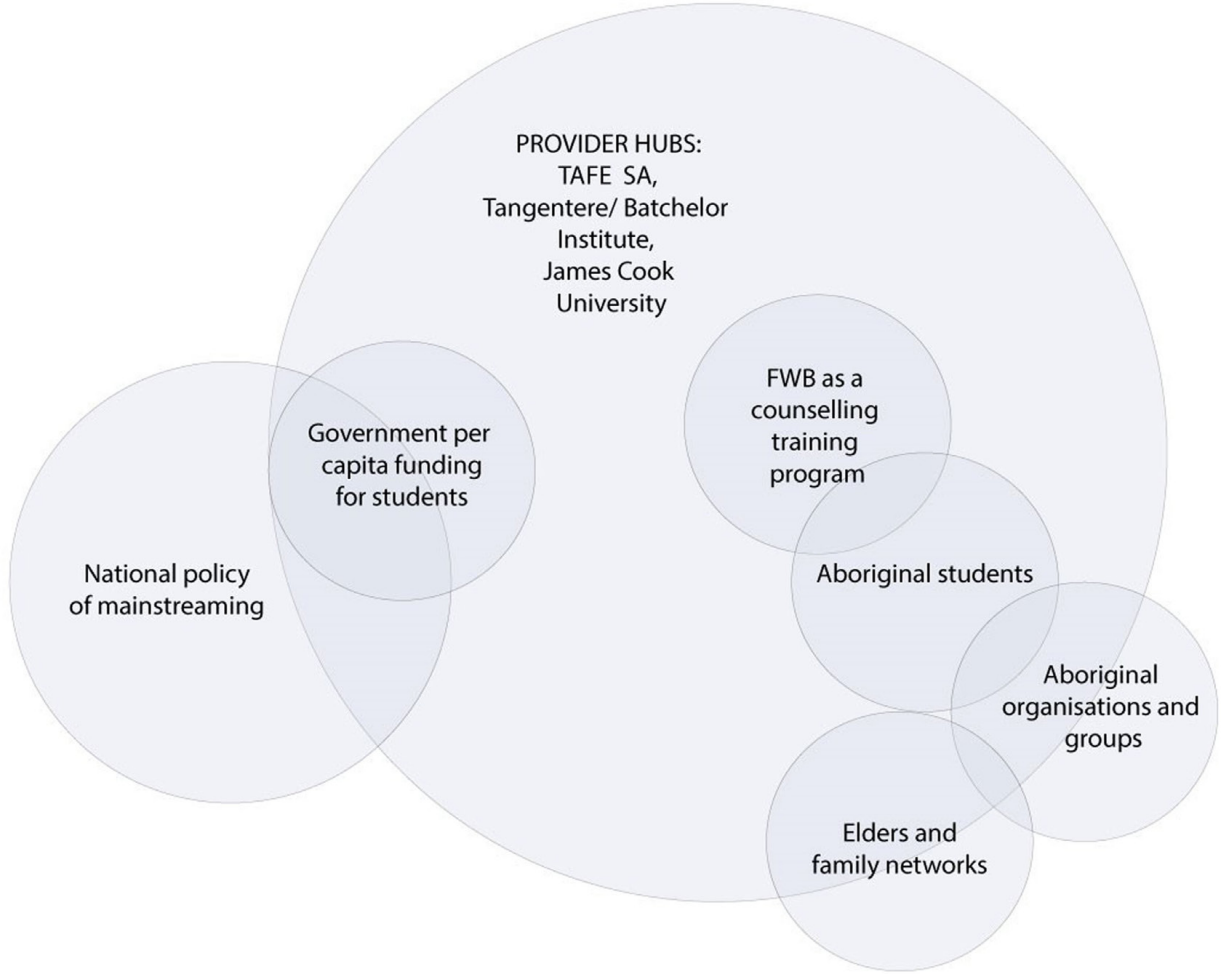
Project map of the social worlds of FWB framed through the arena of training and capacity development (1995–current).

Consistent with the new national policy of mainstreaming, a new phase of FWB development was launched in the guise of an Aboriginal counseling training and capacity development program. Only one key social world was engaged in this arena (Figure 4): the mainstream Technical and Further Education, South Australia (TAFESA), which was administered in the mid-1990s by the same government department as the Branch. FWB was accredited as a certificate II and III course, delivered initially by some of the early FWB agents who were employed as facilitators through the TAFE system and provided Aboriginal leadership.

Technical and Further Education, South Australia operated within the national mainstream environment for training and education (left of Figure 4) that encouraged “a human capital model wherein education is seen to be an investment from which both the individual and ultimately the nation benefit” (35). Thus, the early conceptualization of FWB counseling training was to provide a course for “all Aboriginal people who want to learn how to solve their own personal and family problems *without depending on welfare services*” (36). The benefits of the course for Aboriginal empowerment and leadership was exemplified by two Aboriginal participants who both became program facilitators and remained involved with the program for several years. One said:

[I] made big changes in my life, some sad changes and changes that I never thought that I would make, but it needed to be made…. Since I’ve done the FWB and done a lot of changing and healing in myself, I realise now and appreciate that knowledge is power.

Similarly, the other reflected:

It really opened my eyes in terms of, you know, understanding my own journey. And you know the things that I’ve um—[pause]—endured over the years of my life, and had greater understanding where I was at that time and why.

However, within the counseling training and capacity development arena, the focus shifted from its previous emphasis on individual empowerment to developing Aboriginal students’ capacity within mainstream training organizations for further education and employment in youth work, health and community services (right of Figure 4) (37). Per capita funding for the Aboriginal-specific small-group program now required a guaranteed minimum number of students to make the course viable (left of Figure 4). Within this context, Aboriginal students were recruited through promotion and advertising within communities, job service agencies, employment registers, other courses, schools, and other networks.

Within the training and capacity development arena, FWB retained its interactive, participatory small group delivery style. However, in tying training outcomes to economic measures and the interests of employers and funders, training providers found it challenging to foreground the interests of Aboriginal community organizations. Moreover, attempts to provide transformational Aboriginal-centered education based on ethical principles and terms of reference were subjected to scrutiny and questioning (38–40). Largely missing from the social arena, therefore, was the leading role of community organizations and groups, which had previously determined local foci for the program in response to community-defined priorities.

For Aboriginal students, the program became subject to the challenges common to many education and training programs—levels of attrition for Aboriginal students were significantly higher than was the case for other students, levels of completion markedly lower, and employment outcomes worse for Aboriginal compared to non-Aboriginal course graduates (41, 42). An early South Australian government report, for example, showed that: “many training programs have not produced desired outcomes for Aboriginal people compared with non-Aboriginal people” (43).

Technical and Further Education, South Australia attempted to provide student support through employing Training Support Officers who promoted a supportive case management approach, assessing academic skills and planning educational pathways to assist students reach their desired employment outcomes (37). In practice, however, the levels and extent of student support required, and the reluctance or inability of some students to transition to mainstream courses or employment, proved challenging for TAFE to sustain. A program manager recalled: “trying to keep it together and fighting for funding to run the courses, yeah and getting numbers, and just a one-man band”. TAFE providers’ capacity to consult and partner with community organizations to ensure that the course met students’ needs and expectations was also quite limited (37). The only program adaptation that occurred through this arena was through accreditation and quality improvement processes every 5 years.

Rather than supporting the improvement of extant course quality, governments responded to poor indicators of Aboriginal education and employment outcomes by developing further training initiatives. Aboriginal training courses proliferated and FWB became just one of many Aboriginal-specific training and capacity building programs provided by a plethora of registered training organizations (right side of Figure 4). For example, Hudson (44) identified 36 registered training organizations across Australia that provided training for Aboriginal health workers alone. Nevertheless, demand for FWB has persisted and the accredited course continues to be regular delivered in South Australia and the Northern Territory, and on an *ad hoc* basis in Queensland.

The initial commitment of FWB agents to provide the program in rural and remote communities also became logistically difficult. A non-Aboriginal researcher and advocate explained:

We’re a relatively low resource requirement, really, but we are a resource; our resources are additional resources…. So there either has to be a serious redirection of somebody to do it or new resources that go with it, and that’s always problematic.

Consequently, in the mid-2000s, a consultation report which informed the development of a South Australian strategic framework for social and emotional wellbeing reported that representatives from at least nine communities had requested “community-level healing such as the FWB Program” [(45), p. 55]. However, by then, community healing programs were virtually non-existent in the regions (45). Several Aboriginal health workers also pointed to a need to heal themselves before they could heal their communities and requested FWB delivery of such skills for training themselves and others (45). But given a lack of TAFE or other training facilities in rural and remote areas, combined with the logistical difficulties of coordinating remote training courses, demand outstripped the resources of the provider hubs to deliver. By 2006, TAFESA had established only one additional TAFESA training site—in the South Australian Riverlands (46).

Subsequently, two other provider hubs also established FWB training courses (Figure 3). Supported by TAFESA, from 2007 Batchelor Institute of Tertiary Education in Alice Springs, Northern Territory (both in partnership with Tangentyere Council and independently) also delivered FWB as an accredited Certificate II and III training course. TAFESA facilitators initially traveled to support facilitator training in Alice Springs, with FWB courses later delivered by local facilitators on a needs- or issues-basis with participants recruited as relevant to the issue or need. The James Cook University Empowerment Research Program in Cairns, Queensland, also developed and delivered a 30-h postgraduate training course from 2008. This course was developed with TAFESA permission in response to feedback from community participants (see Arenas three and four) that professionals working with them needed to do FWB. Titled “Empowerment and Change,” the course was adapted from the first stage of FWB with additional theoretical readings and aimed to better skill future Australian health, education and social service workers in the operationalization of values-based empowerment approaches, particularly those working in Aboriginal contexts (47). It was delivered in Cairns and Townsville to students recruited primarily from the social sciences and health disciplines.

All three key provider hubs delivered the FWB training to students at least annually. Thus, the training and capacity development arena fostered sustained delivery of FWB in the three provider sites, but there was limited capacity within the arena to spread the counseling training program to other training providers.

### Arena 3: Aboriginal Social and Emotional Wellbeing Promotion and Empowerment Research (1996–Current)

**Figure 5:**
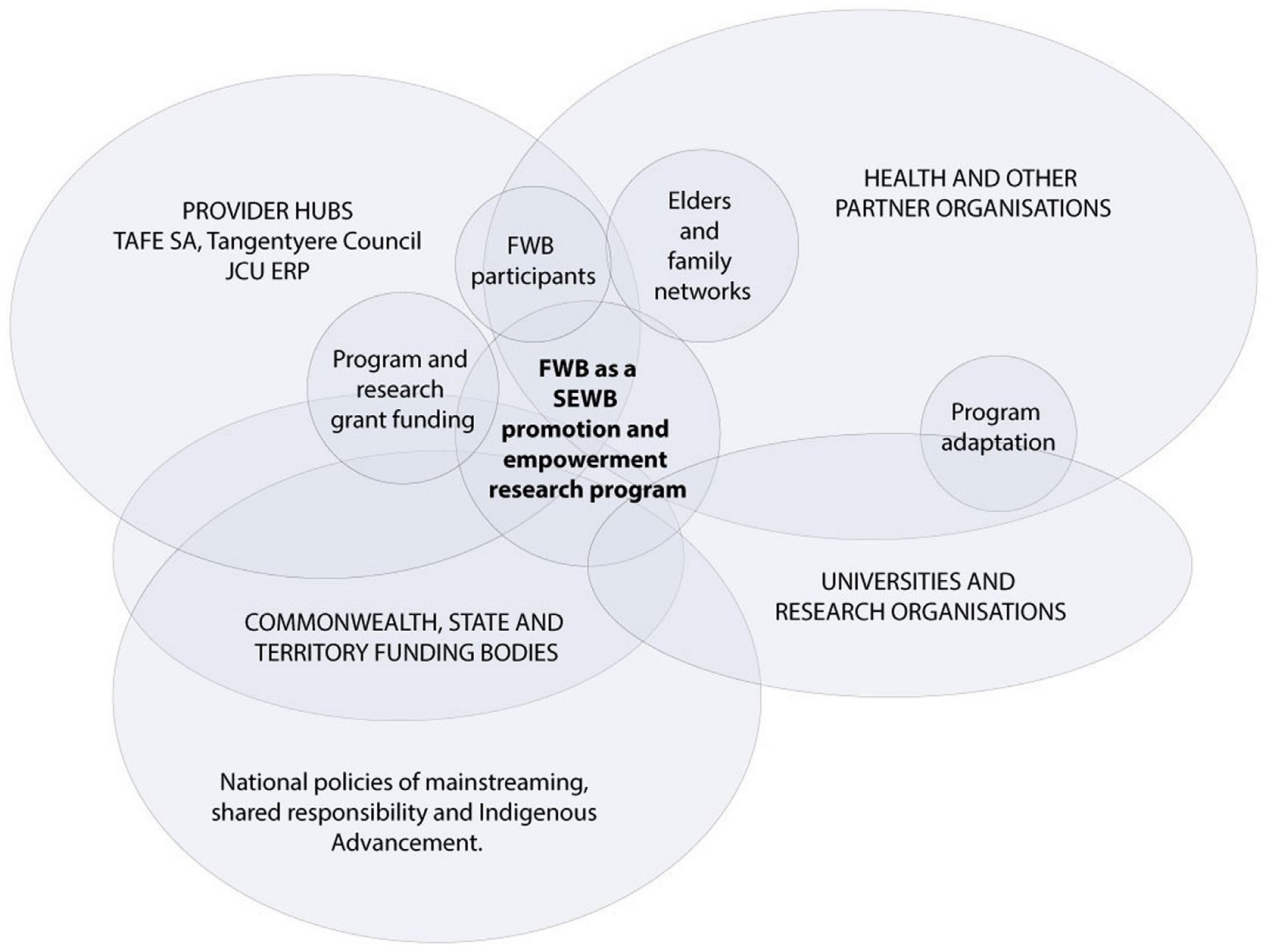
Project map of the social worlds of FWB framed through the arena of SEWB promotion and empowerment research (1996–current).

From 1996, FWB was developed as a social and emotional wellbeing (SEWB) promotion program, with associated empowerment research. In this more complex social arena, four key social worlds interacted through regional innovation hubs to spread the program. The four social worlds engaged in the delivery of FWB as a SEWB promotion and empowerment research program were the three main provider hubs (TAFESA in Adelaide, Tangentyere Council/Batchelor Institute in Alice Springs, and James Cook University in Cairns) and their partner organizations, government funders, and researcher organizations (Figure 5). This arena was driven primarily by FWB agents within the provider hubs (left of Figure 5) and/or partner organizations (right of Figure 5) by writing funding submissions to a broad range of Commonwealth and state government funding bodies (left of Figure 5), usually for short-term pilot programs in single sites. When successful, a provider hub was contracted to deliver the program. FWB provided an empirical foundation for a 10-year phased Empowerment Research Program (right of Figure 5), seeking to understand the relevance of concepts of empowerment and control to the social determinants of Aboriginal health, and in turn, providing program credibility (48). Within this arena, FWB required minimal infrastructure, but short-term funding meant that in most occasions of program delivery, implementation was not sustained beyond the initial pilot programs (29, 49).

The first delivery of FWB within the SEWB promotion and empowerment research arena occurred in 1996 in response to a tragic cluster of youth suicides in Alice Springs. Like other emerging SEWB programs at the time, the program aimed to enhance mental health through moving away from individualized and “problematized” approaches to encompass “domains of health and wellbeing such as connection to land or ‘country,’ culture, spirituality, ancestry, family and community” (50). Building on knowledge of FWB through family networks with AEDB Director Les Nayda (right side of Figure 5), a coalition of Alice Springs community-controlled health organizations successfully submitted a grant to the National Suicide Prevention Strategy for pilot FWB delivery to local health and human services workers. As mentioned in the last section, TAFESA facilitators traveled to Alice Springs to deliver FWB stage one in 1996 through an intensive facilitation of weekly group sessions on a fly-in fly-out basis, and the full five stages in 1998–1999.

This Alice Springs delivery provided an opportunity for the first external evaluation of FWB (right side of Figure 5). Researcher Komla Tsey from Menzies School of Health Research participated in the program and observed its effects, also attaining a qualification by which he could later facilitate the course for others. The evaluation demonstrated the program’s potential for engaging Aboriginal people in ways that were highly relevant to their daily lives. The evaluation also documented the strengths and limitations of FWB including potential to reach other target groups such as men, and to further program needs, such as for dedicated facilitator training and longer term funding to maintain the impetus built during pilot projects (24).

Upon Tsey’s relocation in 2000 to the University of Queensland and later to James Cook University in Cairns, he took the advice of Dr Pat Anderson, current Chairperson of the Lowitja Institute, that:

A number of initiatives to enhance Aboriginal community resilience and well-being have been implemented, including the Family Wellbeing programme…Komla undertook the first evaluation of the Family Wellbeing programme in 1998. I …encouraged him to continue his work on Aboriginal empowerment.

Tsey then established a new focus for FWB as an empirical foundation for a 10-year phased Empowerment Research Program, seeking to understand the relevance of international concepts of empowerment and control (right side of Figure 5) to the social determinants of Aboriginal health (48). In this, he and his team were influenced by evidence that relative powerlessness resulted from colonization and was a major factor in poor Aboriginal health. Yet there was little research about how to foster empowerment, let alone evaluate it (24). One Aboriginal researcher commented:

What people don’t get about it [FWB]… is that it’s not a health program, it’s not an education program. It’s an empowerment program by itself that needs to be used before people engage in other programs. How do you evaluate and say to government or organisations ‘well if you put all this money in prevention then it would save you this cost up here?’

Subsequently, community-controlled organizations in Alice Springs and North Queensland partnered with the James Cook University Empowerment Research Program and Batchelor innovation hubs to build facilitation capacity. The program was sustained through multiple short-term grants (bottom of Figure 5) and a commitment to enable Indigenous people to creatively address their own problems by adapting FWB to different issues.

Family Wellbeing was subsequently framed to respond to a range of SEWB issues identified by community partners. These included stress, loss and grief, suicide prevention (51, 52), family violence (46, 53–55), alcohol addictions (48), fetal alcohol syndrome, anger management, sexual health (56), mental health (57), and prison inmate education. It was also framed as a response for wellbeing-related issues such as men’s health (58, 59), women’s health, first-time motherhood (60), basic life skills for parenting and relationships, and individual or group counseling. The capacity to flexibly adapt FWB to these diverse issues (right side of Figure 5) was facilitated by its competencies, resonant with the capacity to enhance learning and flourishing in a changing and uncertain world (61).

Flexibility was evident also in program delivery, with priority given to meeting demand for locally situated needs. The progam was designed for and delivered primarily by and to Aboriginal people, but it was also delivered to non-Aboriginal participants. Optimally, FWB was delivered to small participant groups of 6–15 participants, but this study found a continuum of group sizes from one-on-one counseling sessions to groups with more than 50 participants. Although the preference of FWB agents was to implement the program through bottom-up spread, there were also examples of FWB deliveries consistent with top-down approaches. These included FWB delivery to prison inmates and alcohol rehabilitation clients and the mandating of attendance by the courts as part of offenders’ parole sentencing.

For example, the north Queensland regional community-controlled health organization, Apunipima Cape York Health Council, was keen to reframe the Certificate II FWB counseling program. They wanted to return to its original intent as a strengths-based community engagement, including empowerment, leadership and community development program and they worked with Tsey to adapt FWB as a two-step approach. A non-Aboriginal manager commented on the step-wise process of implementation in her organization:

So in the first instance it was for my team and then it was adopted corporately… to then extend it out to create partnerships with other regional organisations.

This approach incorporated FWB stage one plus facilitator training, considered sufficient to enable cofacilitation of stage one to others. This provided a small taste of FWB that allowed participants to decide whether they wanted to commit to the full course. It also enabled the spread and sustained implementation of FWB by quickly building facilitation capacity in north Queensland (62).

Returning to the theoretical roots of FWB, the research team trained and supported community-based Aboriginal researchers to use participatory action research processes to guide local efforts to identify and address priorities. For example, in Yarrabah, FWB participants expressed concerns and acted to improve housing, school attendance, the feasibility of establishing small business enterprises and reduce violence. In addition, community-identified needs led to FWB Program delivery to strengthen workforce capacity (63), to build intercultural and interdisciplinary teams (64), and to facilitate organizational change (65, 66). Such efforts built Aboriginal ownership and capacity. Simple incremental evaluation tools were developed to measure their effects, resulting in practical local improvements to support action to further progress community priorities and interests (67). Participatory action research approaches were utilized in diverse situations across north Queensland, but were not taken up by the South Australian or Alice Springs provider hubs.

Another example was the adaptation of FWB for children’s wellbeing and school participation in response to an invitation in 2003 by the Aboriginal principal of two Cape York community state schools. They piloted delivery of an adapted version of FWB to students years five to seven to tackle some of their challenging wellbeing issues. The adaptation included three topics—leadership, basic human needs and relationships—and three class projects—a class plan for a collective change project, a FWB logo competition and a photographic project using disposable cameras for students to explore their identity and connections with family, friends, places, and other things of significance (62). Similar to results for adults, the pilot demonstrated the program’s potential for enhancing the personal growth and empowerment of primary school students (68).

Building on this pilot delivery, a major program adaptation was then undertaken to adapt FWB to address children’s wellbeing, and hence school attendance. In 2006, selected topics of FWB were adapted through the Cape York Bound for Success New Basics Curriculum to develop a “rich task” for Cape York schools. The task, titled Making My Way Through, was targeted specifically at grade seven students as a strategy for preparing them for transitioning to either boarding school or a local high school and aimed to build students’ resilience. It was trialed in two Cape York schools, resulting in improved student attendance and engagement (research respondent, personal communication, May 15, 2010). Delivery of the task was not evaluated and was not sustained in Queensland.

In 2004, a new national policy for Aboriginal development of shared responsibility (bottom left of Figure 5) was introduced. In part, the approach was informed by Aboriginal leaders such as Noel Pearson, who argued for innovative programs that empowered and enabled Aboriginal family groups to take greater control and responsibility for their own situation (69). The whole government approach purportedly aimed to strengthen Aboriginal community capacity to negotiate with governments, and governments’ capacities to address the fragmentation and lack of coordination of programs (70, 71). ATSIC was abolished, with the Commonwealth Government claiming that “the experiment in separate representation, elected representation, for Indigenous people, was a failure” (Howard and Vanstone, 2004 in 34). Shared Responsibility Agreements (SRAs) were introduced which required an Aboriginal community to make certain commitments toward achieving a nominated goal in return for a government commitment of funds or services. Maddison (34) described the SRA arrangements not as partnerships between the government and Aboriginal people, but an attempt by government to address “a problem to be solved” (p. 1). This was a much more top-down approach.

Only two examples of FWB implementation through an SRA with the Australian government were found. In 2005, the Ngangganawili (Wiluna, WA, Australia) Aboriginal Community Controlled Health organization negotiated the delivery of FWB as a leadership program for Aboriginal women. A second SRA signed by the north-eastern Tasmanian Indigenous community and Tasmanian and Australian governments in 2007 built on an earlier Tasmanian FWB delivery and aimed to implement a TAFE course for Aboriginal people to be trained as Family Wellbeing Counselors to assist people affected by family violence (55). With residual ATSIC funding of $22,219 and TAFE Tasmania’s promise of in-kind support (72), TAFE Tasmania contracted an Aboriginal facilitator from TAFESA to deliver the training. The facilitator relocated to Tasmania for 12 months and delivered the Certificate II course to human service workers in Hobart and Launceston, traveling weekly between the two places. Despite training of a group of motivated workers and recognition of an urgent need to deal with family violence issues, the agreement included none of the preconditions for sustainability (a sustained coordinator, facilitation training nor evaluation). An Aboriginal facilitator described the lost opportunity:

I tried endlessly to get into other Aboriginal organisations. I tried endlessly to get into the actual [name] prison. They were very excited about it, but it turned out, ‘no we have no funding’. I just kept getting blocked. And then you give up.

The TAFESA facilitator returned to Adelaide and the FWB was discontinued.

From the late 2000s, FWB was increasingly incorporated as one element of complex multi-strategy programs (73). Researchers collaborated with partner organizations; prioritizing Aboriginal control and capacity strengthening through the research process. In September 2008, for example, a suicide prevention program delivered by the James Cook University Empowerment Research Program in north Queensland incorporated FWB as an engagement, values clarification and capacity building tool as part of a knowledge sharing project between four Aboriginal community men’s support groups. Community-based men’s group leaders were trained in FWB Certificate II and III and supported to deliver the training to men’s group members and others in each of their communities (51, 74). Engagement, planning, implementation and evaluation of such larger scale projects required high levels of organizational capacity and longer timeframes. These increasingly complex projects were managed by the provider hubs and delivered by partner organizations with third-party non-government organizations and private consultancies. Evaluations of such complex programs were also published by researchers from Flinders University, the Australian Institute for Family Studies and the Cooperative Research Center for Aboriginal Health (CRCAH; now Lowitja Institute). Evidence about the empowerment approach built national credibility and external support for FWB, which contributed to further resourcing.

In 2014, the Commonwealth government had adopted yet another national policy (bottom left of Figure 5)—the policy of Indigenous Advancement. IAS replaced more than 150 individual programs and activities with five priority areas: jobs, land and economy; children and schooling; safety and wellbeing; culture and capability and remote strategies. As for the previous policies, however, for the most part, partner organizations continued to apply for funding for single deliveries across single sites. Thus, while the SEWB arena fostered considerable program spread and adaptation, in most sites, delivery was not sustained. A non-Aboriginal researcher and advocate reflected:

[…] to try and get that level of sustainability is quite difficult for a program of the sort that we’re talking about, very difficult really. If you look across public sector programs of this sort of nature, which are non-mainstream, to survive 10 years is quite a challenge, when you’re looking at at least three governments in a period of time like that.

Partner hubs became concerned about short-term approaches to Aboriginal health, wellbeing and development, stringent accountability requirements, and an absence of sustained partnerships with government at high levels (75).

Finally, in 2016, a National Center for FWB was established at James Cook University in Cairns. Funded by Australia’s national Indigenous health research organization, the Lowitja Institute, the National Center was established to bring together FWB training providers, user organizations and communities, researchers and funders, in collaborative partnerships to sustain implementation and evaluation. Key targets for the Center include parents, youth, family support, child safety and child and maternal health workers.

## Discussion

Many Australian indicators show that structural conditions in Australia have generally failed to improve outcomes for the 670,000 Indigenous Australians (3% of population). As a broad indicator of health, the current gap between Indigenous and non-Indigenous Australians’ life expectancy at birth is still estimated to be 10.6 years for males and 9.5 years for females (76). International and Australian evidence of achievement of health targets has shown that it is possible to improve health equality (77, 78). The scaling of promising or proven programs could contribute to healthcare improvement but there is an ongoing need to ensure that the structural conditions enable and provide opportunity rather than constrain scaling.

**Figure 6:**
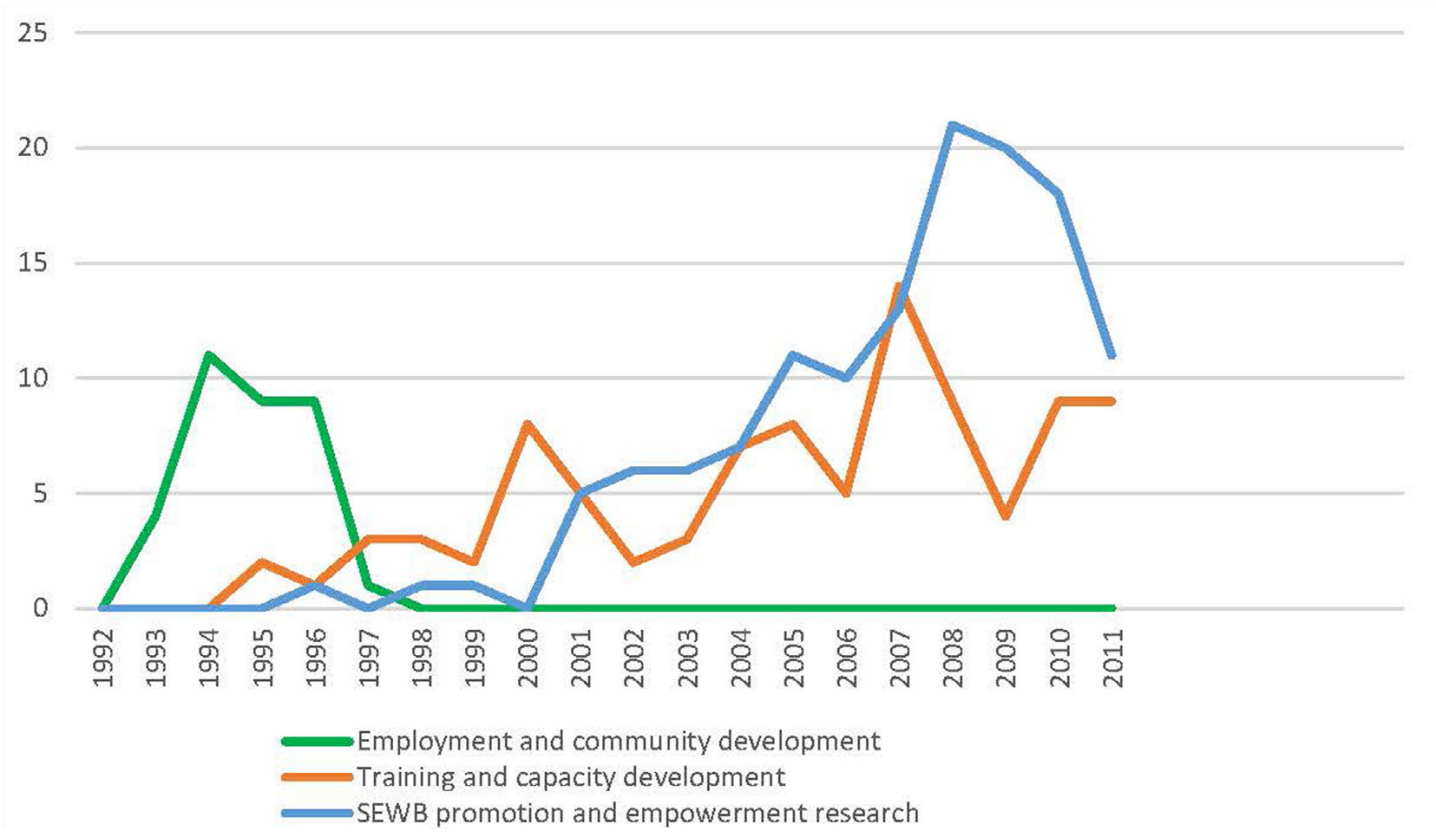
Spread of FWB through social arenas 1993–2011. *Program delivery data were collected only for this period.

The case of FWB demonstrates that scaling up of programs does not occur through a consistent, lineal process (Figure 6). As mentioned in the introduction, the process of program scaling encompassed transfer, implementation, sustainability, shift in ownership and capacity, and adaptation. Over 24 years, FWB was scaled up through three social arenas (Aboriginal employment and community development, Aboriginal training and capacity development, and Aboriginal social and emotional wellbeing promotion and empowerment research) in response to demand from Aboriginal end-users. Across the years, the structural political, ideological, and economic conditions within the three social arenas influenced variations in the processes and outcomes of FWB scaling.

Overarching conditions that fostered program scaling were government policies and the availability of funding support and control; leadership by Aboriginal Elders and others and associated networks; and research evidence that built credibility for the program. These structural conditions were derived from both Aboriginal and Western knowledge domains. Separating the Aboriginal from the Western domain is not clear-cut because both affected program scaling, and FWB offered an opportunity for interaction at the intersection. However, it is important to acknowledge and consider the continuum between these Aboriginal and Western structural conditions, and to acknowledge that the conditions across this continuum influenced interactions and negotiations about the scaling of FWB.

The availability of resources, particularly funding from government programs, was critical to program scaling, particularly the sustainability of implementation. National Aboriginal policies of self-management (1975–1996), mainstreaming (1996–2004), shared responsibility (2004–2014), and Indigenous advancement (2014–current) provided shifting historical frameworks for Aboriginal development, influencing Commonwealth, state and territory governments’ operational policies, which drove the availability of resources and support for programs such as FWB.

For example, the national policy of self-management (1975–1996) supported Aboriginal self-governance processes, whereby Aboriginal people themselves made decisions about long-term goals and objectives for their communities, what kinds of development they wanted and what actions needed to be taken to achieve those goals (79). Control of program funding was in Aboriginal (ATSIC) hands. This allowed FWB originators to develop an ambitious vision of FWB as an evidence-informed employment and community development program, and to transfer, implement and adapt the program across South Australian sites in response to community demand.

The second “training and capacity development” arena was framed in response to the national policy of mainstreaming. Here, FWB agents were able to attract funding to develop the certificate II FWB vocational educational program and to attain sustained government funding in three sites over more than 20 years. In contrast, FWB was funded within the SEWB promotion and empowerment research arena in more than 40 sites by one-off government grants, but implementation was sustained by this arena beyond 5 years in only three sites. As for many programs, Indigenous organizations reported difficulty in sustaining FWB with small, short duration grants with their accompanying administrative and fiscal accountability requirements (8). The average duration of 2000 Indigenous service grants awarded by the 3 largest Commonwealth Government departments through 2010–2011 was 15 months (80). Henry et al. (81) argued that such funding arrangements constituted institutional racism as they severely restrict the capacity of Aboriginal community-controlled services and others to provide culturally secure services. Overall, the scaling of FWB was therefore most active when control of funding was in Aboriginal hands.

Aboriginal leadership, networks and capacity were also important enabling conditions. In the original employment and community development arena, leaders built Aboriginal ownership and capacity, informal family and community networks, capability and control, and achieved sustained implementation. Aboriginal FWB agents (some of whom were Elders and/or community leaders) took lead roles as program developers, coordinators, facilitators, adaptors, researchers and advocates. Their leadership contributed significantly to the credibility of the program with Aboriginal community members and organizations. The capability of Aboriginal organizations and groups to negotiate with governments and integrate FWB delivery within service provision was also important. Aboriginal networks, including and ties to Elders and family members, were also instrumental in spreading awareness of the program and negotiating aspects of scaling. In contrast, spread was restricted in the training and capacity development arena by the limited capacity within mainstream educational systems for shifts in Aboriginal ownership and capacity.

Also different in the third SEWB promotion and empowerment research arena, informal innovation hubs between researchers with provider and partner organizations provided opportunities for dialog and learning across formal and informal networks of FWB agents and program adaptation [e.g., Ref. (47, 56, 60, 68, 82)]. The international literature documents characteristics of such innovation hubs as the similarity of socioeconomic, cultural and other characteristics of change agents and users; shared language, meanings and value systems; credibility; strong interpersonal networks; shared resources; capacity; linkages at an early stage; joint evaluation of the consequences of the initiative; and social and organizational networks (83, 84). “Adopters served as their own change agents” by adapting and developing the program and associated practices to address the local challenges they faced (11, 83, 85). Such dialog produced moderate shifts in Aboriginal ownership and capacity, and considerable adaptation to the needs of different issues, settings, end user groups and ages. The concept of innovation hubs might thus be useful for supporting future opportunities for adaptation such as for online facilitation and delivery.

Finally, research evidence built credibility for the program, particularly through the SEWB promotion and empowerment research arena. Here, funding applications drew upon research evidence to support one-off grant applications. More than 60 empirical evaluation reports and articles documented the effects of FWB on efforts by Aboriginal individuals, families, organizations and communities to exert greater control and influence over many factors affecting their day-to-day lives (10). Motivated by the perceived relevance and credibility of the program as both Aboriginal-developed and evidence-based, partner organizations used the reports and articles to support funding applications, leading to a shift in ownership and capacity.

The three key enabling conditions found in this study are consistent with those found in the scaling of health programs internationally: political commitment by governments, strong community representation, and the availability of decentralized administrative, political and economic support. In the case of FWB, regional innovation hubs played an important role in negotiations across social worlds to scale the program in response to local Aboriginal community demand. This study also noted the importance of research support in building evidence and program credibility. This suggests a need for further evidence of the enabling and constraining factors underlying program sustainability. In particular, the impact of short-term piloting approaches as a response to what are inherently long-term development issues requires evaluation.

## Conclusion

The history of FWB scaling involved the evolution of the program through three interwoven social arenas: employment and community development; training and capacity building; and SEWB promotion and empowerment research. Within this evolution, the program was tailored to meet the needs of participant groups and, in some cases, radically adapted. Despite demand and commitment of end user groups, the sustained implementation of FWB in only 6 of the 60+ sites suggests the difficulties that Aboriginal organizations face in continuing to deliver programs with small, short duration grants. Conditions that enabled scaling were government policies and the availability of funding support and control; leadership by Aboriginal Elders and others and associated networks; and research evidence which built credibility for the program. The continued scaling of such programs requires enhanced support for provider hubs to facilitate negotiations of program transfer and their sustained implementation by committed partner organizations and individuals within these social arenas.

## Ethics Statement

This study was carried out in accordance with the recommendations of James Cook University Ethics Committee with written informed consent from all subjects in accordance with the Declaration of Helsinki. The protocol was approved by the James Cook University Ethics Committee (H 3532).

## Author Contributions

JM designed the study with guidance from RB, KT, and AC. JM collected and analyzed the data with assistance from CB. JM drafted the initial manuscript which was then read and critically revised by RB, CB, KT, and AC. All authors approved the final version to be published and agreed to be accountable for the content of the work.

## Conflict of Interest Statement

The research was conducted in the absence of any commercial or financial relationships that could be construed as a potential conflict of interest.
